# Insomnia in probable migraine: a population-based study

**DOI:** 10.1186/s10194-016-0681-2

**Published:** 2016-10-06

**Authors:** Jiyoung Kim, Soo-Jin Cho, Won-Joo Kim, Kwang Ik Yang, Chang-Ho Yun, Min Kyung Chu

**Affiliations:** 1 Department of Neurology, Bio Medical Research Institute, Pusan National University Hospital, Pusan National University School of Medicine, Busan, Republic of Korea; 2 Department of Neurology, Dongtan Sacred Heart Hospital, Hallym University College of Medicine, Hwaseong, Republic of Korea; 3 Department of Neurology, Gangnam Severance Hospital, Yonsei University College of Medicine, Seoul, Republic of Korea; 4 Sleep Disorders Center, Department of Neurology, Cheonan Hospital, Soonchunhyang University College of Medicine, Cheonan, Republic of Korea; 5 Clinical Neuroscience Center, Department of Neurology, Seoul National University Bundang Hospital, Seongnam, Republic of Korea; 6 Department of Neurology, Kangnam Sacred Heart Hospital, Hallym University College of Medicine, Seoul, Republic of Korea

**Keywords:** Insomnia, Headache, Migraine, Probable migraine, Epidemiology

## Abstract

**Background:**

Insomnia is a common complaint among individuals with migraine. The close association between insomnia and migraine has been reported in clinic-based and population-based studies. Probable migraine (PM) is a migrainous headache which fulfills all but one criterion in the migraine diagnostic criteria. However, an association between insomnia and PM has rarely been reported. This study is to investigate the association between insomnia and PM in comparison with migraine using data from the Korean Headache-Sleep Study.

**Methods:**

The Korean Headache-Sleep Study is a nation-wide cross-sectional survey for all Korean adults aged 19–69 years. The survey was performed via face-to-face interview using a questionnaire on sleep and headache. If an individual’s Insomnia Severity Index score was ≥15.5, she/he was diagnosed as having insomnia.

**Results:**

Of 2695 participants, the prevalence of migraine, PM and insomnia was 5.3, 14.1 and 3.6 %, respectively. The prevalence of insomnia among subjects with PM was not significantly different compared to those with migraine (8.2 % vs. 9.1 %, *p = 0.860*). However, insomnia prevalence in subjects with PM was significantly higher than in non-headache controls (8.2 % vs. 1.8 %, *p < 0.001*). Insomnia Severity Index score was significantly higher in subjects with migraine compared to those with PM (6.8 ± 5.8 vs. 5.5 ± 5.8, *p = 0.012*). Headache frequency and Headache Impact Test-6 score were significantly higher in subjects with migraine and PM with insomnia compared to those without insomnia. Multivariable linear analyses showed that anxiety, depression, headache frequency and headache intensity were independent variables for contributing the ISI score among subjects with PM.

**Conclusions:**

The prevalence of insomnia among subjects with PM was not significantly different compared to those with migraine. Anxiety, depression, headache frequency and headache intensity were related with ISI score in subjects with PM.

## Background

Insomnia and migraine are common complaints among the general population, affecting 10–30 % and 5–15 % of the general population, respectively [1–3]. Both individuals with insomnia and individuals with migraine experience a limitation of functions and a decrease in quality of life(QOL) due to their symptoms [4–6]. Recent studies have revealed that both conditions imposed a huge amount of personal and social burdens [4, 7].

A close association between insomnia and migraine has been described in previous population-based studies [8–16]. In cross-sectional studies, individuals with migraine had an increased risk of having insomnia [odds ratio (OR) = 1.4–2.6] compared to non-migraineurs and individuals with insomnia had a higher risk of having migraine (OR = 1.5–1.7) compared to individuals without insomnia [8, 11–16]. Longitudinal studies also showed an increase risk [relative risk (RR) = 1.7] for having insomnia among individuals with migraine after 11 years [9]. The association was stronger for those with high frequency migraine. Conversely, individuals with insomnia showed an increased risk of having migraine at 11-year follow-up (RR = 1.7) [10].

Probable migraine (PM) is a migrainous headache fulfilling all but one of the criteria for migraine [17]. Probable migraine was reported to be a common subtype of migraine, with a 1-year prevalence rate ranging from 4.3 to 14.6 % according to the previous population-based studies [18–20]. PM is a common headache type for visiting doctors in neurology department [21]. Individuals with PM often experienced headache-related disability and decreased QOL like migraine [18, 19]. Therefore, PM is an important type of headache in clinical practice.

Although insomnia appears to be closely associated with migraine, the association between insomnia and PM has rarely been reported. The Korean Headache-Sleep Study (KHSS) was a population-based survey regarding headache type and sleep and provided an opportunity to evaluate the association between insomnia and PM. In the present study, we 1) described the prevalence of insomnia, migraine and PM across a general population-based sample; 2) compared the prevalence of insomnia between migraine and PM; and 3) assessed the impact of insomnia in clinical characteristics of migraine and PM using the data from KHSS.

## Methods

### Survey

We used data from the KHSS in the present study. The KHSS was a nationwide, cross-sectional survey regarding headache types, symptoms of anxiety and depression, and sleep status among the adult population aged 19–69 years. The study design and methods of KHSS have been previously described in detail [22]. Briefly, we used a 2-stage clustered random sampling method for all Korean territories except Jeju-do, proportional to the population distribution. All interviewers were employees of Gallup Korea and had previously experience with social survey interviewing. The survey was conducted by door-to-door visits and face-to-face interviews from November 2011 to January 2012. The study was approved by the institutional review board/ethics committee of Hallym University Sacred Heart Hospital and was performed in accordance with the ethical standards laid out in the 1964 Declaration of Helsinki and its subsequent amendments [23]. Informed consent was obtained from all participants before each interview.

### Assessment of migraine and PM

Diagnoses of migraine and PM were based on criteria A to D for migraine without aura (code 1.1) in the second edition of the International Classification of Headache Disorders (ICHD-2) [A, at least five attacks; B, attack duration of 4–72 h; C, any 2 of the 4 typical headache characteristics (i.e., unilateral location, pulsating quality, moderate-to-severe pain intensity, and aggravation by or caused by routine physical activity); and D, attacks associated with at least one of the following: nausea, vomiting, or both photophobia and phonophobia] [17]. Subjects who met all of these criteria were considered to have migraine, whereas subjects who met all criteria except one were considered to have PM. Cases with headaches that met the criteria for both PM and tension-type headaches did not receive a PM diagnosis, as per ICHD-2. Because migraine and PM with aura (codes 1.2.1 and 1.6.2, respectively) are difficult to document using the questionnaire method, we did not evaluate aura [24]. Accordingly, these conditions were classified as migraine and PM in the present study.

### Assessment of insomnia

We used the Insomnia Severity Index (ISI) to diagnose and classify insomnia among subjects. The ISI is a brief self-reported questionnaire that measures the patient’s perception of insomnia severity [25]. The ISI is composed of 7 items that evaluate various aspects of insomnia symptoms. Each ISI item was rated on a scale of 0–4. The total ISI score were divided into 4 categories: 0–7, no clinically significant insomnia; 8–14, subthreshold insomnia; 15–21: moderate insomnia; and 22–28: severe insomnia. If a participant’s ISI score was 15.5 or more, she/he was diagnosed as having insomnia. The Korean version of ISI was previously validated with good sensitivity and specificity and showed a 15.5 cut-off score for discriminating patients with insomnia [26].

### Assessment of anxiety and depression

We used the Goldberg Anxiety Scale (GAS) to diagnose anxiety among subjects. This scale comprises four screening items and five supplementary items [27, 28]. Subjects who answered positively for two or more screening items and five or more of all scale items were classified as having anxiety. The Korean version of the scale has an 82.0 % sensitivity and 94.4 % specificity for diagnosing anxiety [28].

The Patient Health Questionnaire-9(PHQ-9) was used to diagnose depression [29]. Subjects who had scores of 10 or more on this measure were considered to have depression. The Korean version of PHQ-9 has an 81.1 % sensitivity and 89.9 % specificity [30].

### Analyses

The Kolmogorov–Smirnov test was used to evaluate the normality of the distribution; after the normality was confirmed, we utilized Student’s t-test and Chi-square test for comparison of prevalence rates where appropriate. If the normality of the distribution was not confirmed, we used Mann–Whitney *U* test. Multiple linear regression analysis with stepwise selection was used to evaluate contributing factors for ISI score among patients with PM. A significance level of *p < 0.05* was set for all analyses. Statistical analyses were performed with the Statistical Package for the Social Sciences 22.0 (SPSS 22.0; IBM, Armonk, NY, USA).

As with most survey sampling designs, there were missing data (resulting from non-response) for several variables. All of the reported results are based on the available data; as such, the total numbers of some variables diverge from 2695 because of missing data for that particular variable. Imputation techniques were not used because we wanted to minimize non-response effects [31].

## Results

### Survey

**Fig. 1:**
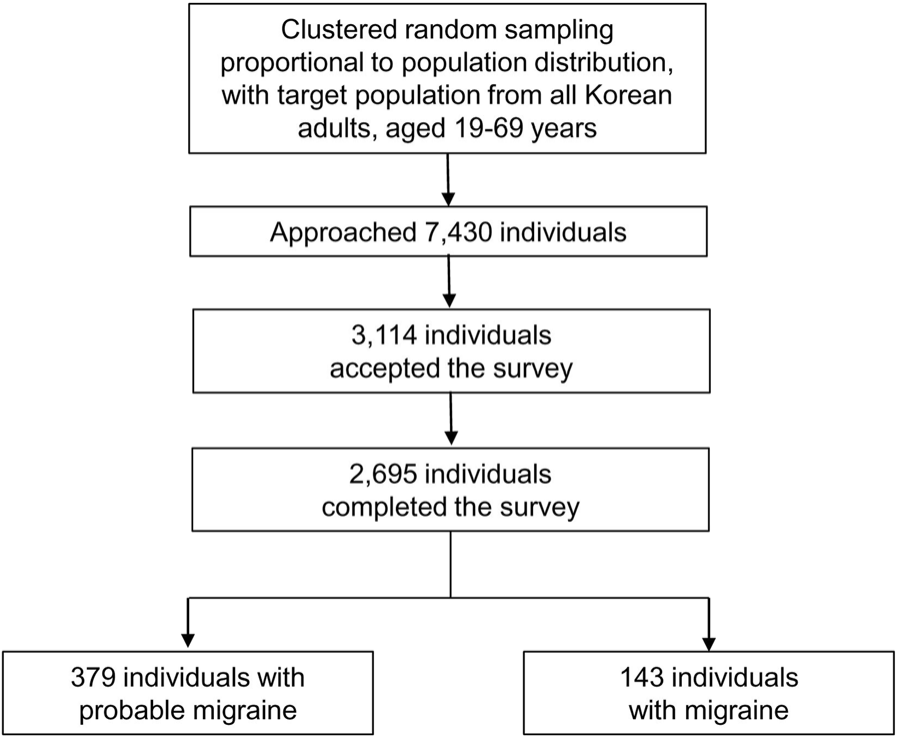
Flow chart depicting the participation of subjects in the Korean Headache-Sleep Study

Interviewers approached 7430 individuals. Although three thousand one hundred and fourteen agreed to participate in the survey (rejection rate 58.1 %), 419 individuals withdrew from participation in the interview. Overall, 2695 subjects completed the survey (cooperation rate 36.3 %; Fig. 1). We found no significant differences in the distributions of age, gender, size of residential area, and educational level from those of the general population of Korea (Table 1).

**Table 1:** Sociodemographic characteristics of survey participants; the total Korean population; and cases identified as having migraine, probable migraine, and insomnia

	Survey participants	Total population	*P-value*	Migraine	Probable migraine	Insomnia
	N (%)	N (%)		N, % (95 % CI)	N, % (95 % CI)	N, % (95 % CI)
Gender						
Men	1345 (49.3)	17,584,365 (50.6)	*0.854* ^*a*^	36, 2.7 (1.8–3.5)	136, 10.1 (8.5–11.8)	33, 2.5 (1.6–3.3)
Women	1350 (50.7)	17,198,350 (49.4)		107, 7.9 (6.5–9.4)	243, 17.9 (15.8–19.9)	83, 4.7 (3.5–5.8)
Age						
19–29	542 (20.5)	7,717,947 (22.2)	*0.917* ^*a*^	25, 4.5 (2.7–6.2)	69, 12.6 (9.8–15.4)	14, 2.6 (1.2–3.9)
30–39	604 (21.9)	8,349,487 (24.0)		42, 7.0 (4.9–9.1)	102, 16.8 (13.7–19.8)	19, 3.1 (1.7–4.5)
40–49	611 (23.1)	8,613,110 (24.8)		39, 6.5 (4.5–8.4)	102, 16.8 (13.9–19.8)	21, 3.4 (2.0–4.9)
50–59	529 (18.9)	6,167,505 (17.7)		22, 4.1 (2.4–5.9)	62, 11.6 (8.8–14.4)	23, 4.3 (2.6–6.1)
60–69	409 (15.6)	3,934,666 (11.3)		15, 3.9 (2.0–5.7)	44, 11.2 (8.1–14.2)	19, 4.6 (2.6–6.7)
Size of residential area						
Large city	1248 (46.3)	16,776,771 (48.2)	*0.921* ^*a*^	76, 6.1 (4.8–7.5)	180, 14.4 (12.4–16.3)	47, 3.8 (2.7–4.8)
Medium-to-small city	1186 (44.0)	15,164,345 (43.6)		48, 4.0 (2.9–5.2)	174, 14.7 (12.7–16.7)	41, 3.8 (2.4–4.5)
Rural area	261 (9.7)	2,841,599 (8.2)		19, 7.4 (4.2–10.6)	25, 9.7 (6.1–13.3)	8, 3.1 (1.0–5.2)
Education level						
Middle school or less	393 (14.9)	6,608,716 (19.0)	*0.752* ^*a*^	22, 5.5 (4.2–7.7)	44, 11.5 (8.4–14.7)	23, 5.9 (3.5–8.2)
High school	1208 (44.5)	15,234,829 (43.8)		60, 5.0 (3.8–6.3)	178, 14.7 (12.7–16.7)	40, 3.3 (2.3–4.3)
College or more	1068 (39.6)	12,939,170 (37.2)		60, 5.6 (4.3–7.0)	155, 14.4 (12.3–16.5)	32, 3.0 (2.0–4.0)
Not responded	26 (1.0)			1, 3.8 (0.0–11.8)	2, 7.7 (0.0–18.7)	1, 3.8 (0.0–11.8)
Total	2695 (100.0)	34,782,715 (100.0)		143, 5.3 (4.5–6.2)	379, 14.1 (12.7–15.4)	96, 3.6 (2.9–4.3)

*CI* Confidence Interval

^a^Comparison of gender, age group, size of residential area, and educational level distributions between the sample in the present study and the total population of Korea

### Prevalence of migraine, PM and insomnia

Of the 2695 subjects, 143 (5.3 %) were classified as having migraine and 379 (14.1 %) were classified as having PM during the previous year. Among participants, 333 (12.4 %) had subthreshold insomnia, 83 (3.1 %) had moderate insomnia and 26 (1.0 %) had severe insomnia. Ninety six (3.6 %) reported ≥ 15.5 ISI score and were classified as having insomnia (Table 1).

### Prevalence and severity of insomnia among subjects with migraine and PM

**Fig. 2:**
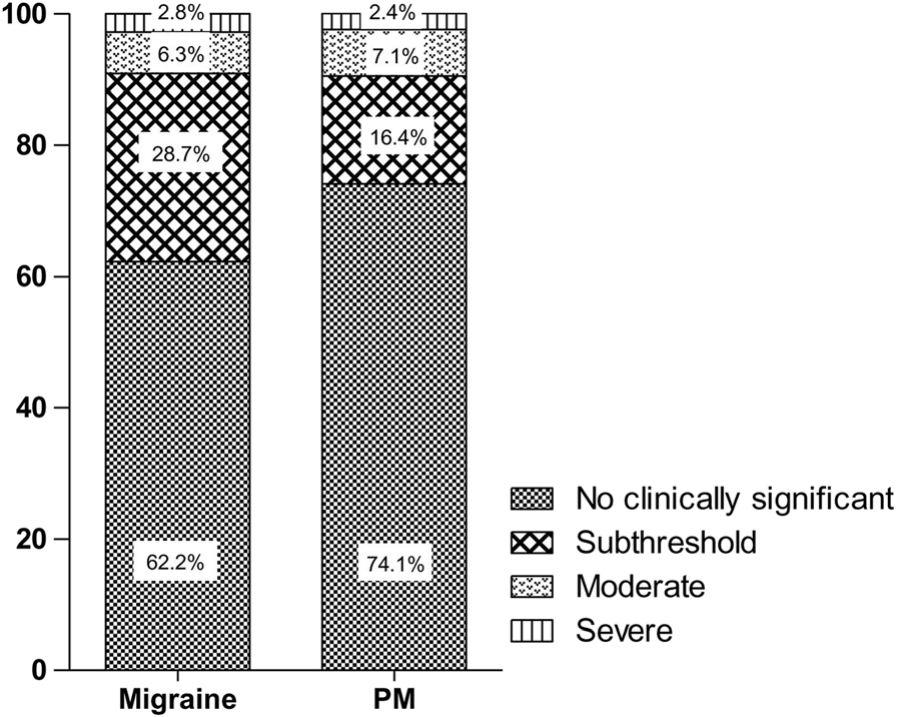
Distribution of insomnia severity in subjects with migraine and probable migraine

Thirteen (9.1 %) subjects with migraine and 31 (8.2 %) subjects with PM were diagnosed as having insomnia. The prevalence of insomnia among subjects with PM was not significantly different compared to those with migraine (*p = 0.860*). Compared to non-headache controls (1.8 %), the prevalence of insomnia among subjects with migraine [OR = 5.6, 95 % confidence interval (CI) = 2.8–11.2, *p < 0.001*] and PM (OR = 5.0, 95 % CI = 2.9–8.5, *p < 0.001*) was significantly higher. Subjects with migraine had higher ISI score compared to subjects with PM (6.8 ± 5.8 vs. 5.5 ± 5.8, *p = 0.012*). The proportion of severe insomnia (2.8 % vs. 2.4 %, *p* = 0.782) and moderate insomnia (6.3 % vs. 7.1 %, *p* = 0.739) was not significantly different between migraine and PM. However, the proportion of subclinical insomnia was higher among subjects with migraine compared to those with PM (28.7 % vs. 16.4 %, *p* = 0.002) (Fig. 2).

### Demographic and clinical characteristics of subjects with migraine and PM according to the presence of insomnia

Subjects with migraine and PM who had insomnia showed significantly higher GAS score and PHQ-9 score compared to those without insomnia. However, sociodemographic variables such as age, proportion of gender, size of residential and education level were not different according to the presence of insomnia (Table 2).

**Table 2:** The difference of demographic and clinical variables in subjects with migraine and PM according to insomnia status

	Migraine			PM		
	Insomnia (+), *N* = 13	Insomnia (−), *N* = 130	*p*	Insomnia, *N* = 31	Insomnia, *N* = 348	*p*
Age, years	43.3 ± 16.3	41.0 ± 12.0	*0.653* ^*a*^	45.0 ± 13.2	41.6 ± 12.5	*0.151*
Female, n (%)	10 (76.9 %)	97 (74.6 %)	*0.855*	21 (67.7 %)	222 (63.8 %)	*0.702*
Size of residential area			*0.857*			*0.382*
Large city, n (%)	7 (53.8 %)	69 (53.1 %)		14 (45.2 %)	166 (47.7 %)	
Medium-to-small city, n (%)	5 (38.5 %)	43 (33.1 %)		13 (41.9 %)	161 (46.3 %)	
Rural city, n (%)	1 (7.7 %)	18 (13.8 %)		4 (12.9 %)	21 (6.0 %)	
Education level			*0.081*			*0.886*
Middle school or less, n (%)	6 (46.2 %)	16 (12.3 %)		3 (9.7 %)	4 (11.8 %)	
High school, n (%)	3 (23.1 %)	57 (43.8 %)		16 (51.6 %)	162 (46.6 %)	
College or more, n (%)	4 (30.8)	56 (43.1 %)		12 (38.7 %)	143 (41.1 %)	
No answer, n (%)	0 (0.0 %)	1 (0.8 %)		0 (0.0 %)	2 (0.6 %)	
GAS score	6.4 ± 2.1	3.3 ± 2.3	*<0.001* ^*a*^	6.6 ± 1.8	2.4 ± 2.1	*<0.001*
PHQ-9 score	12.3 ± 6.4	4.0 ± 4.2	*<0.001* ^*a*^	12.3 ± 6.7	2.8 ± 3.1	*<0.001*

*PM* Probable migraine, *GAS* Goldberg Anxiety Scale, *PHQ-9* The Patient Health Questionnaire-9, *SD* Standard deviation

Values are expressed as mean ± SD, ^a^Mann-Whitney *U* test

### Headache characteristics of migraine and PM according to the presence of insomnia

The median and 25–75 % interquantile range values of the headache frequency, Visual Analog Scale (VAS) score for headache intensity and Headache Impact Test-6 (HIT-6) score were shown in Table 3. Subjects with migraine and PM who had insomnia showed significantly higher headache frequencies per month [migraine: 4.0, (2.0–11.0) vs. 1.0 (0.3–4.0), *p = 0.004*; PM: 2.0 (0.4–4.0) vs. 1.0 (0.3–2.0), *p = 0.004*] and HIT-6 scores [migraine: 65.0 (59.5–71.5) vs. 52.0 (47.0–60.0), *p < 0.001*; PM: 54.0 (48.0–62.0) vs. 45.5 (42.0–52.0), *p < 0.001*] compared to those without insomnia. Subjects with PM who had insomnia showed significantly higher VAS score for headache intensity [PM: 6.0 (5.0–7.0) vs. 5.0 (4.0–6.0), *p < 0.001*) compared to those without insomnia, but subjects with migraine who had insomnia [migraine: 7.0 (5.5–8.5) vs. 6.0 (5.0–7.0), *p = 0.076*] did not show significantly different VAS score compared to those without insomnia.

**Table 3:** Headache frequency, headache pain intensity (VAS score), and Headache Impact Test-6 score in subjects with migraine or PM according to insomnia status

	Migraine			PM		
	Insomnia (+), *N* = 13	Insomnia (−), *N* = 130	*p*	Insomnia (+), *N* = 31	Insomnia (−), *N* = 348	*p*
	Median (25–75 %)	Median (25–75 %)		Median (25–75 %)	Median (25–75 %)	
Headache frequency^a^ (per 1 month)	4.0 (2.0–11.0)	1.0 (0.3–4.0)	*0.004*	2.0 (0.4–4.0)	1.0 (0.3–2.0)	*0.004*
VAS score for headache intensity^a^	7.0 (5.5–8.5)	6.0 (5.0–7.0)	*0.076*	6.0 (5.0–7.0)	5.0 (4.0–6.0)	*<0.001*
HIT-6 score^a^	65.0 (59.5–71.5)	52.0 (47.0–60.0)	*<0.001*	54.0 (48.0–62.0)	45.5 (42.0–52.0)	*<0.001*

*PM* Probable migraine, *VAS* Visual Analogue Scale*, HIT-6* Headache Impact Test-6

^a^Mann-Whitney *U* test

### Multiple linear regression analyses for contributing factors for ISI score in subjects with PM

We performed the multiple linear regression analyses of clinical variables including sociodemographic variables (age, gender, size of residential area, educational level), GAS score, PHQ-9 score, headache frequency and VAS score to evaluate the contributing factors for ISI score (Table 4). The strongest contributing factor was PHQ-9 score (β = 0.530, *p < 0.001*), followed by GAS score (β = 0.268, *p < 0.001*), headache frequency (β = 0.119, *p < 0.001*) and VAS score (β = 0.076, *p = 0.026*) for headache intensity.

**Table 4:** Analysis of contributing factors related to the ISI score in subjects with PM

	Unstandardized coefficients		Standardized coefficients	t	*P*	Tolerance	VIF
	B	Standard error	Beta				
PHQ-9 score	0.695	0.054	0.530	12.876	<0.001	0.643	1.556
GAS score	0.654	0.103	0.268	6.316	<0.001	0.607	1.646
Headache frequency	0.124	0.035	0.119	3.537	<0.001	0.956	1.046
VAS score for headache intensity	0.249	0.111	0.076	2.240	0.026	0.940	1.063

*ISI* Insomnia severity index, *PM* Probable migraine, *GAS* Goldberg Anxiety Scale, *PHQ-9* The Patient Health Questionnaire-9, *VAS* Visual Analogue Scale

R^2^ = 0.592, R^2^_adj_ = 0.588

## Discussion

The main findings in the present study are as follows: 1) The prevalence of migraine, PM and insomnia in the Korea population was 5.3, 14.1 and 3.6 %, respectively; 2) The prevalence of insomnia among subjects with PM (8.2 %) was not significantly different compared to that among subjects with migraine (9.1 %); 3) Headache frequency and HIT-6 score of subjects were higher among migraine and PM subjects with insomnia compared to those without insomnia.

The 1-year prevalence of migraine (5.3 %) in the present study was lower than in previous studies from European (10–25 %) and North American (9–16 %) countries [1]. However, the 1-year prevalence of migraine in Asian countries was 4.7 to 9.1 %, which were lower compared to that in European and North American studies [32]. The migraine prevalence in our study was similar to previous studies in Asian countries.

The 1-year prevalence rate of PM was 14.1 % in the present study. Previous epidemiological studies have reported 1-year prevalence rates of PM ranging from 4.3 to 14.6 %. Thus, the prevalence rate of PM in the present study was similar to those in the previous studies [18, 20, 33–36].

The prevalence of insomnia (≥15.5 ISI score) in the present study was 3.6 %. Insomnia prevalence has been previously reported, ranging from 15.3 to 22.8 % in Asian countries including Korea [37–39]. These findings are inconsistent with the present study. This may reflect different insomnia diagnostic criteria. Most previous population-based studies about insomnia probed insomnia symptoms by asking whether subjects had difficult falling asleep, staying asleep and waking too early in the morning. A previous population-based study reported prevalence of insomnia symptoms at least three nights per week was 17.0 %, but insomnia disorder prevalence based on Diagnostic and Statistical Manual of Mental Disorders (DSM)-IV criteria was 5.0 % in this study [39].

Although the prevalence rate of insomnia was not significantly different between subjects with migraine and PM, the ISI score was significantly different between the two groups in the present study. The difference was associated with the higher prevalence of subthreshold insomnia (8 ≤ ISI score ≤ 14) among subjects with migraine compared to those with PM. The prevalence rate of insomnia in subjects with migraine and PM was higher compared to non-headache controls in the present study. The finding agrees with findings in previous studies concerning the association between headache and insomnia [3]. Cross-sectional studies showed that headache or migraine had increased ORs compared to non-headache controls and longitudinal studies showed bidirectional comorbidity of two conditions: subjects with insomnia showed an increased risk for headache at 11-year follow up and vice versa [10, 40].

A few studies have addressed the relationship between PM and insomnia among children. Children with adult-like migraine had a higher prevalence of sleep disorders compared to non-headache controls (disorders of initiating and maintaining sleep; migraine 37.1 % vs controls 9.2 %, disorders of arousals; migraine 59.3 % vs controls 10.2 %) [41]. Sleep disorders in infancy could be good predictors for the development of headache [42].

In the present study demonstrated that depression (PHQ-9 score), anxiety (GAD-7 score), headache frequency and headache intensity (VAS score) were independently associated with insomnia (ISI score) among individuals with PM (Table 4). This finding was similar to findings in the previous studies among migraineurs. A study including 78 migraineurs and 208 healthy controls revealed that sleep quality and headache frequency was significantly associated after adjusting anxiety and depression [43]. Another study demonstrated that migraineurs had more sleep problems than healthy controls even after adjusting lifetime anxiety and depression [44].

In the present study, headache frequency and HIT-6 score were higher in migraine and PM subjects with insomnia than in those without insomnia. Since a high headache frequency is one risk factor for chronification of headaches, our finding suggests that insomnia may be associated with the chronification of migraine and PM. We also noted that insomnia was associated with a higher HIT-6 score in subjects with migraine and PM. This finding may imply an increased burden of migraine and PM with the presence of insomnia [45]. Physicians should evaluate accompanying insomnia in subjects with migraine and PM. Careful investigation of the main causes of insomnia including psychiatric co-morbidity is also necessary.

This study has some limitations. First, although the questionnaire in this study was validated for migraine, it was not specifically validated for PM. However, the questionnaire itself was based on ICHD-2 and PM was classified based on the ICHD-2 diagnostic criteria. Second, although this study is population-based and had low sampling error, its statistical power was limited in terms of examining the subgroups. In other words, some results might not have reached statistical significance in subgroup analysis because of the limited sample size.

Despite these limitations, our study has several strengths. First, our study was based on clustered random sampling proportional to the Korean population distribution with low sampling error. This condition allowed us to accurately investigate the prevalence of insomnia, migraine, and PM among the Korean adult population. Second, this study explored the relation between PM and insomnia. This relation has been rarely studied. Third, we investigated both anxiety and depression, which are common comorbidities among subjects with insomnia and migraine, and assessed the effect of anxiety and depression in the association of insomnia with migraine and PM. Balancing the limitations and strengths, we believe that the present study accurately describes the association between insomnia and PM in comparison with migraine.

## Conclusions

The prevalence rate of insomnia in individuals with PM is not significantly different compared to those with migraine. Insomnia aggravates headache symptoms among individuals with PM as well as among individuals with migraine.

## Abbreviations

CI: Confidence interval
DSM-IV: Diagnostic and Statistical Manual of Mental Disorders-IV
HIT-6: Headache Impact Test-6
ICHD: International Classification of Headache Disorders
ISI: Insomnia severity index
KHSS: Korean Headache-Sleep Study
OR: Odds ratio
PHQ-9: Patient Health Questionnaire-9
PM: Probable migraine
QOL: Quality of life
GAS: Goldberg Anxiety Scale
RR: Relative risk
VAS: Visual Analogue Scale.
